# Histological response and blood glucose level in a diabetic animal model after the oral administration of *Mucuna pruriens*: A systematic review and meta-analysis

**DOI:** 10.14202/vetworld.2025.1377-1388

**Published:** 2025-05-31

**Authors:** Tri Wahyu Pangestiningsih, Dian Meididewi Nuraini, Morsid Andityas, Ariana Ariana

**Affiliations:** 1 Department of Anatomy, Faculty of Veterinary Medicine, Universitas Gadjah Mada, Yogyakarta, 55281, Indonesia; 2 Department of Animal Science, Faculty of Animal Science, Universitas Sebelas Maret, Surakarta, 55361, Indonesia; 3 Veterinary Technology Study Program, Department of Bioresources Technology and Veterinary, Vocational College, Universitas Gadjah Mada, 55281, Indonesia

**Keywords:** blood glucose, diabetes mellitus, herbal medicine, histology, meta-analysis, *Mucuna pruriens*

## Abstract

**Background and Aim:**

*Mucuna pruriens* (MP) has emerged as a promising natural antidiabetic agent due to its rich bioactive composition. Although numerous preclinical studies have reported its hypoglycemic and histological benefits, a comprehensive synthesis quantifying these effects has been lacking. This study systematically evaluated the dual impact of orally administered MP extract on histopathological changes and blood glucose levels in diabetic animal models through a systematic review and meta-analysis.

**Material and Methods:**

A systematic literature search was conducted across four databases (PubMed, Scopus, ScienceDirect, and Google Scholar) without date restrictions. Eligible *in vivo* studies were selected based on predefined inclusion criteria, and data were extracted following PRISMA guidelines. Risk of bias was assessed using the systematic review center for laboratory animal experimentation tools. Histological outcomes were summarized descriptively, while blood glucose levels were analyzed quantitatively using a random-effects meta-analysis. Subgroup analyses were performed based on MP concentration, duration of administration, and plant part used.

**Results:**

Sixteen studies were included, with 13 eligible for meta-analysis. MP extract significantly reduced blood glucose levels, with an overall standardized mean difference of −18.36 (95% confidence intervals: −21.22, −15.51; p < 0.01). Subgroup analyses revealed that lower MP doses (≤100 mg/kg) achieved superior glycemic control with prolonged administration (>4 weeks), whereas higher doses (≥200 mg/kg) were most effective within 1–4 weeks. Histological analysis indicated regenerative effects of MP on the pancreas, liver, pituitary gland, and corpus cavernosum. Seed extracts exhibited a stronger hypoglycemic effect compared to leaf extracts. Potential publication bias was detected but was addressed through trim-and-fill analysis.

**Conclusion:**

MP extract demonstrates significant antidiabetic potential through glycemic regulation and organ tissue restoration. Lower concentrations are preferable for long-term administration, while higher concentrations are optimal for short-term therapy. The findings advocate MP as a valuable candidate for integrative diabetes management strategies. Further clinical studies are recommended to validate its translational potential.

## INTRODUCTION

Diabetes mellitus has emerged as a significant global health threat in recent years. In 2021, the International Diabetes Federation reported that approximately 537 million adults were affected by diabetes, with projections indicating an increase to more than 700 million by 2045 [1]. Diabetes mellitus is broadly categorized into two main types: Type 1 diabetes, accounting for approximately 5%–10% of cases, and Type 2 diabetes, which is more prevalent and often associated with obesity and metabolic syndrome [2]. Current therapeutic approaches primarily involve oral hypoglycemic agents and insulin therapy, including agents such as metformin, sulfonylureas, thiazolidinediones, as well as newer classes such as dipeptidyl peptidase-4 inhibitors and sodium-glucose cotransporter-2 inhibitors [3]. These medications are effective in regulating glycemic control and managing body weight and cardiovascular function [4, 5]. However, adverse reactions, such as hypoglycemia and diabetic ketoacidosis, are commonly associated with these therapies, necessitating vigilant monitoring for adverse drug reactions [6].

In parallel with conventional pharmacotherapy, considerable interest has emerged in alternative diabetes treatments utilizing herbal supplements. *Mucuna pruriens* (MP) is a leguminous plant that thrives in tropical and subtropical regions [7]. It possesses a high protein concentration (23%–25%) and good digestibility, rendering it a valuable alternative food source [8]. Moreover, MP is rich in diverse bioactive compounds, including levodopa (L-DOPA), flavonoids, and other phytochemicals, which contribute to its therapeutic properties, particularly in the context of diabetes [9]. While previous studies by Majekodunmi *et al*. [10], Bhaskar *et al*. [11], and Reuben-Kalu *et al*. [12] have highlighted the hypoglycemic effects of MP seed extract, this study is the first to systematically differentiate therapeutic outcomes based on various plant parts using meta-analytic comparisons. Furthermore, the antidiabetic effects of MP are not limited to its seeds but extend to other parts, such as the leaves [13, 14]. Beyond glycemic regulation, MP extracts have also been reported to confer protection and promote cellular regeneration of organ structures compromised under diabetic conditions [12, 15–17]. These regenerative effects are primarily attributed to the antioxidant activities of MP’s bioactive compounds, which mitigate oxidative damage and facilitate cellular recovery [12].

Despite extensive preclinical evidence supporting the antidiabetic potential of MP, most studies have independently evaluated either its hypoglycemic effects or histological improvements without integrating both outcomes systematically. In addition, the comparative efficacy of different plant parts, such as seeds versus leaves, has not been rigorously synthesized across experimental models. Previous studies have predo-minantly focused on short-term glycemic outcomes, often overlooking the influence of treatment duration and dosage on the long-term therapeutic potential of MP. Furthermore, the heterogeneity in experimental designs, including variations in animal models, extra-ction methods, and diabetes induction protocols, has complicated the establishment of standardized therapeutic guidelines. Consequently, a critical gap remains regarding the comprehensive understanding of MP’s pharmacological profile, its time-dependent efficacy, and the specific plant parts conferring the most pronounced antidiabetic effects.

The present study aims to systematically evaluate the effects of orally administered MP extract on histological features and blood glucose levels in diabetic animal models through a systematic review and meta-analysis. Specifically, this study seeks to (1) consolidate evidence of MP’s dual impact on glycemic regulation and histopathological restoration, (2) compare the therapeutic outcomes between different plant parts (seeds vs. leaves), (3) assess the influence of dosage and treatment duration on its efficacy, and (4) provide a foundational synthesis to guide future experimental designs and potential clinical translation. By addressing these objectives, this study intends to offer novel insi-ghts into optimizing the therapeutic application of MP for diabetes management.

## MATERIALS AND METHODS

### Ethical approval

Ethical approval was not required for this study. A systematic literature review was conducted following the Preferred Reporting Items for Systematic Reviews and Meta-Analysis (PRISMA) guidelines [18].

### Study period and location

This systematic review and meta-analysis included a comprehensive search conducted on February 2, 2024, with no restrictions on publication date or geographical location.

### Study registration

The protocol for this systematic review and meta-analysis was prospectively registered with the Open Science Framework (OSF) [https://osf.io/bkhg6/], ensuring methodological transparency and reproducibility.

### PICOS framework

The inclusion and exclusion criteria for this review were determined based on the PICOS framework:

- Population (P): Animal models with experimentally induced diabetes mellitus.
- Intervention (I): Oral administration of MP extract from any plant part.
- Comparator (C): Diabetic control groups without MP treatment.
- Outcomes (O): Changes in blood glucose levels and histopathological improvements in target organs.
- Study design (S): I*n vivo* experimental studies.

### Search strategy

Literature searches were conducted using a combination of predefined keywords such as “*Mucuna pruriens*,” “velvet beans,” “diabetes,” “diabetic,” and “animal,” combined using Boolean operators “AND” and “OR.” These keywords were entered into four international databases: PubMed, Scopus, Science-Direct, and Google Scholar. The search was limited to peer-reviewed studies published in English or Indonesian. Sample size and publication date were not limited. All literature retrieved from these databases was collated and uploaded into Rayyan-Intelligent Systematic Review (https://www.rayyan.ai/) for initial screening [19].

### Inclusion criteria

This review uniquely focuses on *in vivo* experi-mental studies of monotherapy administration of MP extract, ensuring an unconfounded assessment of its histological and metabolic impacts. On the contrary, studies with *in vitro* or *ex vivo* models, non-animal studies, combination interventions with substances other than MP extract, review studies, protocols, theses, conference abstracts, and posters were excluded from the study.

### Data extraction and quality assessment

Two independent reviewers screened the retri-eved studies, and discrepancies between the two revi-ewers were resolved through discussion. After the initial screening, the full texts of the included studies were further screened, subjected to quality assessment, and used for data extraction.

The quality of the studies included in this research was thoroughly evaluated using the systematic review center for laboratory animal experimentation (SYRCLE) risk of bias (RoB) tool [20]. The assessment involved examining ten domains:

1. Description of the random sequence.
2. Similarity between groups.
3. Animal allocation.
4. Post-randomization bias.
5. Concealment of animal allocation.
6. Description of results.
7. Blinding of results.
8. Friction bias.
9. Outcome bias.
10. Reporting bias (including other biases).

Each domain was rated using one of the following options: “yes,” “no,” or “unclear.” These responses indicate a low RoB, a high RoB, or insufficiently reported details.

Data from the included studies were extracted into a pre-designed Excel® (Microsoft Corp, Redmond, WA, USA). The extracted data included: author name, year, country, species of animal, part of the MP plant used for extraction, concentration of the extract, sample size in each group, diabetes induction method, histological findings, and blood glucose level before and after treatment.

Diabetes was assessed using parameters such as blood glucose level, glycated hemoglobin (HbA1c), and serum insulin concentration. Among the reported biomarkers, blood glucose levels were most consistently measured across studies, allowing for robust cross-study effect size estimation – a methodological strength often lacking in prior reviews. Therefore, blood glucose level was used as the primary parameter for diabetes determination in this study.

### Histological response

The changes in the organs of the diabetic control and treated groups were summarized descriptively to evaluate the effect of MP administration on the asses-sed organs.

### Meta-analysis

All statistical analyses were conducted using R software version 4.3.0 (R Foundation for Statistical Computing, Vienna, Austria), employing the “meta” (v8.0-2) and “metafor” (v4.8-0) packages for meta-analysis procedures [21].

The primary outcome measure was the change in blood glucose levels before and after oral administration of the MP extract in diabetic animal models, expressed as the standardized mean difference (SMD) with corres-ponding 95% confidence intervals (CI). A random-effects model (DerSimonian–Laird estimator) was used to account for expected heterogeneity among studies due to differences in animal species, MP concentrations, diabetes induction methods, and treatment duration.

The means, standard deviations, and sample sizes were extracted for each study. Where data were presented as standard errors, values were converted to standard deviations.

Heterogeneity was assessed using the I^2^ statistic, with values of 25%, 50%, and 75% representing low, moderate, and high heterogeneity, respectively. p < 0.10 for the Cochrane Q-test was considered indicative of statistically significant heterogeneity.

To explore sources of heterogeneity, subgroup analyses were performed based on the following factors:

1. MP extract concentration (≤100 mg/kg, 200 mg/kg, >200 mg/kg).
2. Duration of administration (<1 week, 1–4 weeks, >4 weeks).
3. Plant part used (seed vs. leaf).
4. Diabetes induction method (e.g., alloxan and streptozotocin).

Where sufficient data were available, combinatorial subgroup analyses (e.g., concentration × duration) were conducted to determine interaction effects on outcome variability.

### Assessment of publication bias

Publication bias was assessed using funnel plot asymmetry, Egger’s regression test, and Begg’s rank correlation test, with p < 0.05 suggesting potential bias [22]. Where bias was indicated, a trim-and-fill analysis was performed to estimate the number of missing studies and to recalculate the adjusted overall effect size. The threshold for statistical significance for all two-tailed tests was p < 0.05, except where otherwise specified for heterogeneity or bias assessments.

## RESULTS

### Literature search results

**Figure 1:**
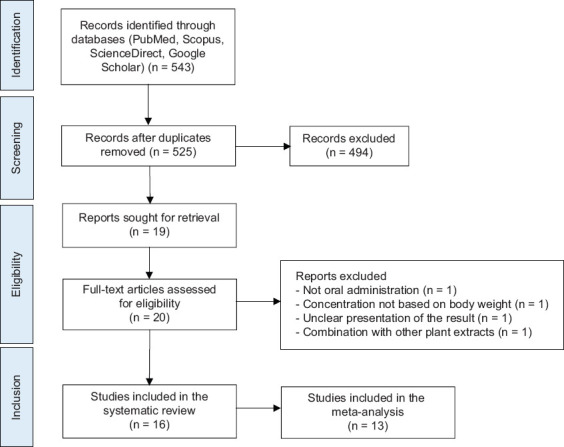
Flow chart detailing the selection process for the meta-analysis on *Mucuna pruriens* extract oral administration effect to the histology features and blood glucose levels.

In total, 543 studies from four databases (PubMed: 26, Scopus: 53, ScienceDirect: 263, Google Scholar: 365) were retrieved and used for primary screening based on titles and abstracts. The primary screening resulted in the exclusion of 525 studies due to duplication and irrelevant study focus. Twenty studies were further screened using full-text evaluation. Based on detailed screening, four studies were excluded due to various reasons, such as the intervention not being orally administered, concentration not being based on body weight, unclear presentation of results, and use of combination extracts. After full-text screening, 16 studies were eligible for inclusion in the systematic review, and 13 studies were included in the meta-analysis. A flowchart detailing the screening process is presented in Figure 1.

### Included study characteristics

Table 1 presents the characteristics of the individual studies included in this systematic review and meta-analysis [10–14, 16, 17, 23–31]. Five studies reporting histopathological features of organs under diabetic conditions were incorporated to summarize histopathological effects before and after the adminis-tration of MP extract. Of these five studies, three reported both blood glucose levels and histopathological features [12, 15, 16], while two studies reported only histopathological features [17, 23]. Thirteen eligible studies with appropriate data on blood glucose levels were included in the meta-analysis. Among these, five studies [12, 13, 15, 24, 25] presented the results using standard error, while the others reported standard deviation. Where standard error was reported, it was converted to standard deviation for analysis.

**Table 1:** The included study characteristics

No.	Author	Country	Diabetes induction	Species	Plant type	Concentration	Sample/group	Total duration
1	Majekodunmi *et al*. [10]	Nigeria	Alloxan	Rat	Seed	5 mg/kg	6	12 weeks
						10 mg/kg		
						20 mg/kg		
						30 mg/kg		
						40 mg/kg		
						50 mg/kg		
						100 mg/kg		
2	Bhaskar *et al*. [11]	India	Streptozotocin	Rat	Seed	100 mg/kg	6	3 weeks
						200 mg/kg		
3	Reuben-Kalu and Renuka [12]	India	Streptozotocin	Rat	Seed	200 mg/kg	6	3 weeks
						400 mg/kg		
4	Eze *et al*. [13]	Nigeria	Alloxan	Rat	Leaves	100 mg/kg	3	3 weeks
						200 mg/kg		
						400 mg/kg		
5	Uchenna *et al*. [14]	Nigeria	Alloxan	Rat	Leaves	200 mg/kg	6	2 weeks
						400 mg/kg		
						600 mg/kg		
6	Rajesh *et al*. [16]	India	Streptozotocin	Rat	Seed	200 mg/kg	6	3 weeks
7	Agbai *et al*. [17]	Nigeria	Alloxan	Rat	Leaves	400 mg/kg	5	3 weeks
						800 mg/kg		
8	Suresh and Prakash [23]	India	Streptozotocin	Rat	Seed	200 mg/kg	6	60 days
9	Murugan *et al*. [24]	India	Alloxan	Rat	Leaves	250 mg/kg	6	1 week
10	Bhadra *et al*. [25]	Bangladesh	Oral glucose	Rat	Seed	50 mg/kg	6	120 min
						100 mg/kg		
						200 mg/kg		
11	Rathi *et al*. [26]	India	Alloxan	Rat	NA	200 mg/kg	8	4 weeks
12	Grover *et al*. [27]	India	Streptozotocin	Mice	NA	200 mg/kg	6	40 days
13	Kar et al. [28]	India	Alloxan	Rat	Seed	250 mg/kg (twice/day)	1	1 week
14	Bhaskar *et al*. [29]	India	Streptozotocin	Rat	Seed	100 mg/kg	6	3 weeks
15	Owa *et al*. [30]	Nigeria	Alloxan	Rat	Seed	100 mg/kg	5	15 days
16	Rathi *et al*. [31]	India	Alloxan Streptozotocin	Rat	NA	100 mg/kg	8	15 weeks
						200 mg/kg		
						400 mg/kg		

The majority of studies were conducted in India (n = 10), followed by Nigeria (n = 5), and one study was conducted in Bangladesh. Eight studies used alloxan to induce diabetes, and six used streptozotocin. A study by Rathi *et al*. [26] used both alloxan and streptozoto-cin, while another used oral glucose [25]. Almost all studies used rats as experimental animals, except for one study that used mice [27].

Regarding the source of MP extract, nine studies used the seeds, four studies used the leaves, and three studies did not specify the plant part used. The study durations varied, ranging from <1 day to 16 weeks, and concentrations of MP extract ranged from 5 to 600 mg/kg body weight.

### Quality assessment

**Figure 2:**
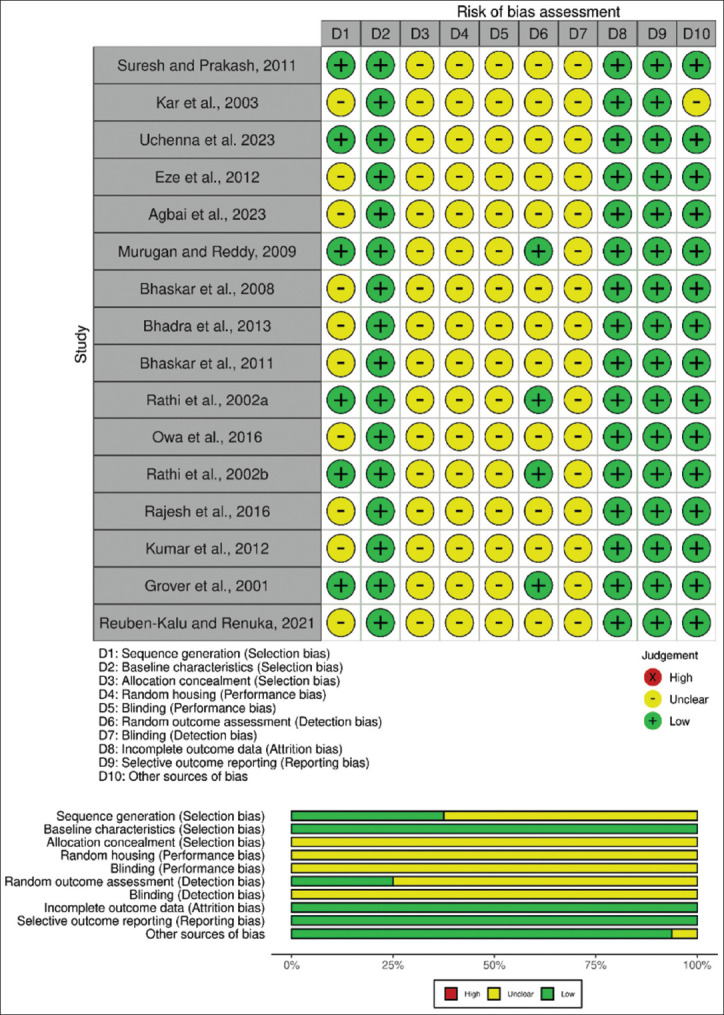
Results of systematic review center for laboratory animal experimentation’s risk of bias assessment for all included studies.

Based on the SYRCLE RoB assessment, the overall quality of included studies was considered moderate, with a prevalence of “yes” answers of 43% and “unclear” answers of 57%. All studies demonstrated a low risk of selection bias concerning similarity between groups, incomplete outcome reporting, and outcome bias (Figure 2). However, assessment items related to animal allocation, post-randomization bias, concealment of animal allocation, and blinding of results were classified as unclear across all studies. Only six studies [14, 23, 24, 26, 27, 31] demonstrated a low RoB with a “yes” answer in at least five indicators. A high RoB was identified in the study by Kar *et al*. [28], which received only three “yes” answers in the quality assessment.

### Histological response

Five included studies [12, 15, 16, 23, 32] reported the effect of MP extract administration on the cellular structure of several organs (Table 2) [12, 15, 16, 23, 32]. The pancreas was the most frequently examined organ, being discussed in three studies [12, 16, 32].

**Table 2:** The histological respond to the administration of oral *Mucuna prurien* extract in diabetic-induced rats.

No.	Author	*Mucuna pruriens* concentration	Organ	Histological features of the diabetic control group	Histological treatment group
1.	Reuben-Kalu *et al*. [12]	200 mg/kg	Pancreas	Histological features showed the degeneration of endocrine cells in the pancreatic islet.	Pancreas endocrine cells regenerated.
		400 mg/kg	Pancreas		The pancreas endocrine cells regenerated better than the dose 200 mg/kg of MP extract treatment.
2	Kumar *et al*. [15]	6 mg/2 kg feed	Liver	Histology of the liver lobules of obese rats shows fatty degeneration of hepatocytes in the peripheral and middle zones. The sinusoid well defined, located between the hepatic muralium.	The liver showed the restoration of hepatic lobules with a decrease in fatty degeneration of hepatocytes in the peripheral and middle zones.
		12 mg/2 kg feed	Liver		The liver showed an increase in hepatic lobule restoration as shown by a small number of fat-vacuolated hepatocytes.
3.	Rajesh *et al*. [16]	200 mg/kg	Pancreas	Histological features showed the karyolysis of the nuclei of the cells in the islet and acini as a characteristic of necrosis. The blood vessels in the islet became congested. Infiltration of inflammatory cells surround the islet.	The number of necrotic cells in the islet decreased, and some cells showed clear, round nuclei. There was no congestion in the blood vessels as a sign of regeneration in the islet. The same histological features of regeneration were also observed in the acini.
		200 mg/kg	Liver	Histological examination of the hepatic lobule architecture became unclear: not defined central veins, hepatocytes structure not in radial arrangement since the destruction of sinusoids, necrosis of hepatocytes, and infiltration of inflammatory cells.	Extracts of MP treatment caused restoration of the hepatic lobule architecture: well defined central veins with hepatocytes arranged radially and sinusoid located between the hepatic muralium, and decreased inflammatory cell counts.
4.	Suresh and Prakash [23]	200 mg/kg	Corpus cavernosum	Histological features showed changes in cavernosal structure, including sinusoidal endothelium wall thickening, blood cell aggregation, the presence of macrophages in the sinusoidal endothelium, and smooth muscle degeneration.	The thickening of sinusoidal endothelium and blood cell aggregation reduced in the supplementation of 200 mg/kg MP.
5.	Agbai *et al*. [32]	400 mg/kg	Pituitary glands	The pituitary glands showed hemorrhage in the adenohypophyse area, necrosis of acidophil cells, and degeneration of cells from moderate to severe.	Glucophage treatment regenerated the pituitary glands. Acidophilic and basophilic cells were well defined. By 400 mg/kg of MP leaf extract treatment, there was a moderate level of cell regeneration, except for basophil cells where a moderate level of athropy was found. Acidophils were more dominant than basophil cells.
		800 mg/kg	Pituitary glands		The pituitary glands showed mild regeneration with moderate basophilic cell atrophy, necrotic cells, and a difficult cell membrane to define. Regeneration at mild levels of pituitary gland cells was found along with atrophy of basophilic cells at moderate levels and some necrotic cells.
		400 mg/kg	Pancreas	Fatty acid degeneration and necrosis in severe level of acini pancreas.	Glucophage treatment regenerated the pancreas cells to a moderate level. Some area showed hemorrhage. After 400 mg/kg MP leaf extract treatment, the cells of pancreatic acini regenerated at a moderate level, and it was difficult to define the membrane cells.

MP: *Mucuna pruriens*

In general, the administration of MP extract provided better histological outcomes compared to positive control groups in all studies, indicating improvements in pancreatic cell structures.

### Overall meta-analysis

The overall meta-analysis yielded a pooled SMD of −18.36 (95% CI: −21.22, −15.51). The heterogeneity test indicated a high level of heterogeneity with an I^2^ value of 92% and p < 0.01.

Various durations, concentrations, and plant parts were used for oral MP supplementation across the included studies. The administration durations ranged from <24 h to 16 weeks. For the meta-analysis, the durations were categorized into three groups: <1 week, 1–4 weeks, and 4 weeks.

Based on subgroup analysis, the administration of MP extract significantly reduced blood glucose levels across all duration groups, with greater reductions observed with prolonged administration (p < 0.01). Administration for >4 weeks resulted in the greatest reduction with an SMD of −29.81 (95% CI: −34.77, −24.86). Administration between 1 and 4 weeks showed an SMD of −8.35 (95% CI: −10.41, −6.29). Administration for <1 week yielded an SMD of −4.73 (95% CI: −6.28 and −3.18) (p < 0.01).

Three concentration groups were defined: ≤100 mg/kg, 200 mg/kg, and >200 mg/kg. Concentration-based subgroup analysis similarly revealed a significant reduction in blood glucose levels across all groups (p < 0.01). Among them, the ≤100 mg/kg concentration group showed the highest reduction with an SMD of −23.18 (95% CI: −27.12, −19.23), compared to 200 mg/kg (−7.19 [95% CI: −9.39, −4.99]) and >200 mg/kg (−9.51 [95% CI: −13.65, −5.37]).

**Figure 3:**
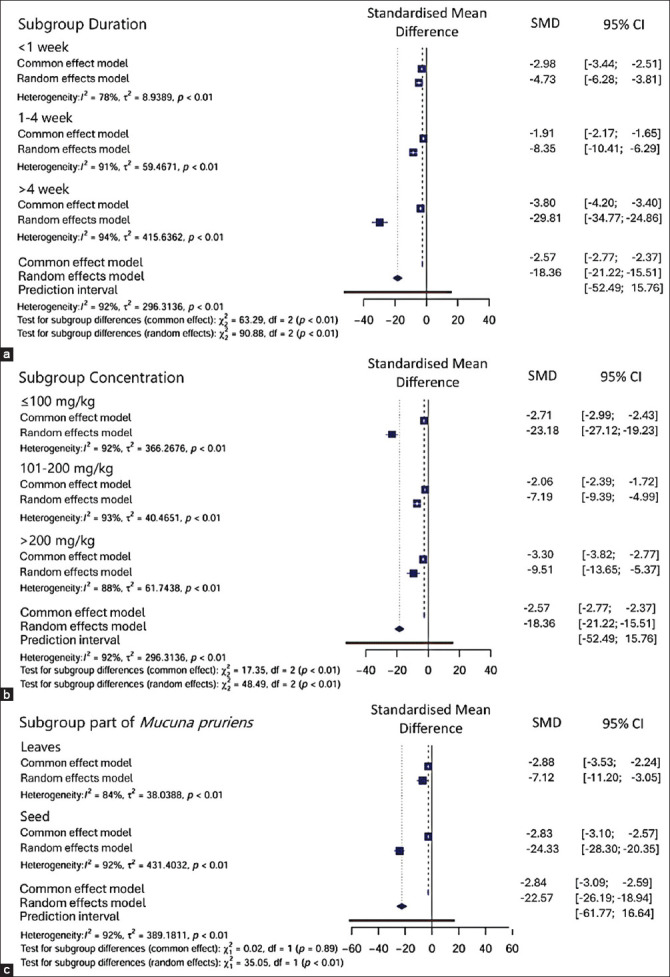
(a-c) Forest plots of the meta-analysis on different subgroups.

Importantly, differential efficacy was observed between the plant parts used: Seed extracts exhibited a greater reduction (SMD: −24.33 [95% CI: −28.30, −20.35]). Compared to leaf extracts (SMD: −7.12 [95% CI: −11.20, and −3.05]) (p < 0.01), a distinction is rarely emphasized in previous literature and highlighted through subgroup analysis (Figure 3).

### Subgroup meta-analysis based on concentration and duration

A further subgroup analysis was conducted to explore sources of heterogeneity based on different concentrations and administration durations (Table 3).

**Table 3:** Subgroup meta-analysis based on the concentration and duration of *Mucuna pruriens* administration in diabetic rats

Concentration	Duration	Standardized mean difference	95% confidence intervals	I^2^	p-value	p-value subgroup
≤100 mg/kg	<1 week	−2.65	−3.60, 1.70	55	0.04	<0.01
	1–4 weeks	−7.66	−9.96, −5.36	91	<0.01	
	>4 week	−38.03	−43.38, −32.69	91	<0.01	
200 mg/kg	<1 week	−4.60	−6.47, −2.74	74	<0.01	0.05
	1–4 weeks	−17.08	−29.00, −5.16	93	<0.01	
	>4 week	−7.38	−10.61, −4.15	94	<0.01	
>200 mg/kg	<1 week	−11.40	−18.37, −4.44	90	<0.01	0.01
	1–4 weeks	−42.68	−81.84, −3.52	89	<0.01	
	>4 week	−7.02	−15.09, 1.06	85	<0.01	

In the low-dosage group (≤100 mg/kg): Short-term administration (<1 week) did not result in significant blood glucose reduction (SMD: −2.65, 95% CI: −3.60, 1.70; p = 0.04). However, prolonged administration (>4 weeks) resulted in a marked reduction (SMD: −38.03, 95% CI: −43.38, −32.69; p < 0.01).

In contrast, administration of higher doses (200 mg/kg or more) resulted in significant reductions in blood glucose levels at all administration durations, with the greatest effects observed during the 1–4 week period. Specifically: Administration of 200 mg/kg showed an SMD of −17.08 (95% CI: −29.00 and −5.16). Administration of >200 mg/kg showed an SMD of −42.68 (95% CI: −81.84 and −3.52) (both p < 0.01).

These results indicate that lower concentrations (≤100 mg/kg) provide better outcomes for long-term therapy, while concentrations ≥200 mg/kg are most effective within 1–4 weeks, with diminished effects observed after prolonged administration.

### Risk of publication bias

**Figure 4:**
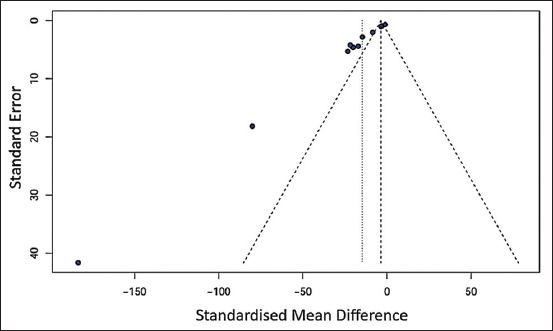
The funnel plot of the publication bias asses-sment showed an asymmetrical graph.

The risk of publication bias was assessed for the overall effect size of oral MP extract administration. Egger’s test and Begg’s test both indicated potential publication bias (p < 0.05), which was also visually evident in the funnel plot (Figure 4).

A trim-and-fill analysis was performed, suggesting the need to add six hypothetical studies to achieve funnel plot symmetry. After the addition, the recalcu-lated SMD was −4.67 (95% CI: −23.10 and 13.75).

## DISCUSSION

### Therapeutic efficacy of MP in glycemic regulation

The antidiabetic potential of MP has been explored in various studies, indicating its efficacy in managing blood glucose levels. The present study demonstrated a significant reduction in blood glucose levels across various concentrations, with superior efficacy observed from seed extracts compared to leaf extracts. These findings align with ethnopharmacological surveys identifying MP among more than 1,200 medicinal plants used for diabetes management due to their hypoglycemic properties [12]. The meta-analysis results further emphasize the effectiveness of lower concentrations (≤100 mg/kg) following prolonged administration. Conversely, concentrations of 200 mg/kg or higher produced optimal effects when administered for 1–4 weeks, after which efficacy declined. This stratified efficacy pattern suggests that MP can be tailored for either short-term glycemic correction or long-term metabolic modulation, thereby contributing a novel dimension to antidiabetic dosing strategies.

### Mechanisms underpinning antidiabetic action

The antidiabetic mechanisms of MP are multi-factorial. One of the principal mechanisms involves the inhibition of alpha-amylase, a digestive enzyme that breaks down carbohydrates. MP has been shown to inhibit alpha-amylase activity, thereby reducing glucose absorption in the intestine [33]. Moreover, its phytochemical constituents – including levo-dopa (L-DOPA), flavonoids, and other secondary meta-bolites – exert antioxidant effects that further enhance antidiabetic activity by attenuating oxidative stress [23]. Oxidative stress is a key pathophysiological mechanism in diabetes, driven by elevated blood glucose levels that promote reactive oxygen species (ROS) generation through mitochondrial dysfunction [34]. Increased ROS levels impair antioxidant defenses, damage pancre-atic tissues, and inhibit insulin production, thereby aggravating hyperglycemia [35, 36]. The antioxidant compounds in MP, particularly L-DOPA, possess free radical scavenging capacity that mitigates oxidative injury and may ultimately reduce diabetes complications [9]. Flavonoids also enhance insulin sensitivity and facilitate glucose uptake in peripheral tissues, contributing to improved glycemic control [37].

### Regenerative and cytoprotective properties

This review is the first to collectively highlight MP’s cytoprotective effects in both endocrine and exocrine tissues, reinforcing its potential as a regenerative agent beyond glycemic modulation. The current systematic review demonstrated improvement in structural integrity of the pancreas, liver, and pituitary gland following MP administration under diabetic conditions [12, 15, 16, 32]. Reuben-Kalu *et al*. [12], Rajesh *et al*. [16], and Agbai *et al*. [32] reported histological recovery in pancreatic tissues following MP extract administration at a dose of 200 mg/kg, suggesting that MP may facilitate pancr-eatic beta-cell regeneration – a critical process in restoring insulin production. Moreover, MP treatment was associated with histological improvement in the liver, pituitary glands, and corpus cavernosum, under-scoring its organ-protective effects in diabetes-induced tissue damage [15, 16, 23, 32]. These regenerative properties are especially important in the context of chronic diabetes, as they address the underlying pathophysiology rather than merely managing clinical symptoms.

### Broader physiological impacts

Beyond glycemic control, MP also affects other physiological parameters pertinent to diabetes management. Several studies have shown that MP can improve lipid profiles [38], with hypocholesterolemic effects that support cardiovascular health in diabetic individuals. In addition, MP exhibits antimicrobial properties, offering protective benefits to diabetic patients who are at increased risk of infections due to immune compromise [39]. These antimicrobial effects are particularly valuable given that infections exacer- bate diabetic complications and pose challenges in clinical management. Notably, MP has also been reported to exert aphrodisiac effects and promote reproductive tissue health, offering potential benefits for addressing diabetes-associated sexual dysfunction – an often under-recognized complication [23, 32]. The multifaceted role of MP in modulating glycemia, lipid metabolism, immune defense, and reproductive health enhances its therapeutic value in comprehensive dia-betes care.

### Safety considerations

The safety profile of MP has also been critically examined. Several studies included in this review evaluated acute toxicity, and none reported adverse effects at the concentrations and durations applied [10, 12, 31]. Furthermore, existing literature supports the general safety of MP for human consu-mption at therapeutic doses [40]. Nonetheless, some studies have identified the presence of toxic consti-tuents in MP, necessitating proper detoxification protocols. This highlights the importance of traditional preparation methods and dosage control to ensure maximal therapeutic benefit with minimal risk [41].

### Limitations

Despite the comprehensive nature of this review and meta-analysis, several limitations should be acknowledged. First, the overall quality of included studies was moderate, with many studies demonstrating unclear or high RoB due to insufficient reporting of randomization procedures, allocation concealment, and blinding. This compromises internal validity and may have introduced methodological heterogeneity. Second, substantial inter-study variability in experimental design – such as differences in animal species, diab-etes induction protocols, extract preparation methods, plant parts used, and treatment durations – may have influenced effect size estimates and contributed to the high heterogeneity (^I^2 = 92%) observed in the meta-analysis. Third, although blood glucose level was selected as the primary outcome due to its consistent reporting, other relevant metabolic parameters such as insulin concentration, HbA1c, and lipid profile were not uniformly reported across studies, limiting a broader understanding of MP’s metabolic impact. Fourth, while the subgroup analyses provided valuable insights into concentration- and duration-dependent effects, the limited number of studies per subgroup may have reduced statistical power. Fifth, publication bias was detected, as evidenced by funnel plot asymmetry and significant Egger’s and Begg’s test results, suggesting potential overestimation of MP’s efficacy. Finally, the absence of clinical trials precludes direct translation of these findings to human populations, underscoring the need for well-designed translational studies.

## CONCLUSION

This systematic review and meta-analysis provide strong evidence for the antidiabetic potential of MP in experimental animal models. Oral administration of MP extract significantly reduced blood glucose levels, with a pooled SMD of −18.36 (95% CI: −21.22 and −15.51) and demonstrated histological improvement in diabetes-induced tissue damage, particularly in the pancreas, liver, and pituitary gland. Subgroup analyses further revealed that seed extracts had greater efficacy than leaf extracts, and that glycemic control was optimized at lower concentrations (≤100 mg/kg) with prolonged administration, while higher concentrations (≥200 mg/kg) were more effective within 1–4 weeks.

These findings underscore the practical value of MP as a multifunctional plant-based agent capable of regulating blood glucose and supporting tissue regeneration. Its therapeutic potential is supported by multiple pharmacological mechanisms, including antioxidant activity, alpha-amylase inhibition, and insulin-sensitizing effects. The strengths of this study include protocol registration, comprehensive meta-analytic modeling, and integration of both biochemical and histopathological outcomes, offering a robust synthesis of MP’s efficacy profile.

Future research should focus on standardizing extract formulations, elucidating molecular mecha-nisms, and conducting well-designed clinical trials to assess efficacy, safety, and dosing parameters in human populations. Overall, MP holds considerable promise as a natural and integrative candidate for diabetes management and metabolic restoration.

## AUTHORS’ CONTRIBUTIONS

TWP: Planned the study, screening process, data analysis, and drafted the manuscript. DMN: Study selection, screening process, data analysis, and drafted the manuscript. MA: Data analysis, interpretation of results, and revised the manuscript. AA: Interpreted the results and revised the manuscript. All authors have read and approved the final manuscript.
